# Doubly Robust Estimation of Optimal Dynamic Treatment Regimes

**DOI:** 10.1007/s12561-013-9097-6

**Published:** 2013-07-12

**Authors:** Jessica K. Barrett, Robin Henderson, Susanne Rosthøj

**Affiliations:** 1 MRC Biostatistics Unit, Institute of Public Health, University Forvie Site, Robinson Way, Cambridge, CB2 0SR UK; 2 School of Mathematics and Statistics, University of Newcastle, Newcastle upon Tyne, UK; 3 Department of Biostatistics, Institute of Public Health, University of Copenhagen, Copenhagen, Denmark

**Keywords:** Causal inference, Dynamic treatment regimes, G-estimation, Regret-regression

## Abstract

We compare methods for estimating optimal dynamic decision rules from observational data, with particular focus on estimating the regret functions defined by Murphy (in J. R. Stat. Soc., Ser. B, Stat. Methodol. 65:331–355, 2003). We formulate a doubly robust version of the regret-regression approach of Almirall et al. (in Biometrics 66:131–139, 2010) and Henderson et al. (in Biometrics 66:1192–1201, 2010) and demonstrate that it is equivalent to a reduced form of Robins’ efficient g-estimation procedure (Robins, in Proceedings of the Second Symposium on Biostatistics. Springer, New York, pp. 189–326, 2004). Simulation studies suggest that while the regret-regression approach is most efficient when there is no model misspecification, in the presence of misspecification the efficient g-estimation procedure is more robust. The g-estimation method can be difficult to apply in complex circumstances, however. We illustrate the ideas and methods through an application on control of blood clotting time for patients on long term anticoagulation.

## Introduction

A dynamic treatment regime is a decision rule, or set of decision rules, which determines how a treatment should be assigned to a patient over time. Typically a patient is observed at regular intervals, and at each visit a treatment decision or *action* *A* is made in response to measurements of *state* *S* taken at that visit together with the history of previous decisions and measurements. An optimal dynamic treatment regime is one which maximises an overall *outcome* *Y* measured at the end of a sequence of visits.

Since the seminal work of Murphy [15] there has been growing interest in biostatistical applications of decision rule methodology. Recent work includes Arjas and Saarela [1], Dawid and Didelez [6], Moodie et al. [13], Zhao et al. [21, 22]. The focus of most work has been on testing for treatment effects, typically for binary *A* and with rather few measurement times. Even in very simple circumstances there can be severe statistical challenges in this area (Chakraborty et al. [3]; Hernan et al. [10]; Moodie and Richardson [14]; Zhang et al. [20]).

Motivated by an application on anticoagulation, we suppose the treatment decision *A* is essentially continuous rather than categorical, and our interest is in estimation of optimal decisions rather than testing. We concentrate on the regret functions proposed by Murphy [15], which are defined in Sect. 2 and form a particular case of the so-called advantage learning class of approaches. A variety of methods have been proposed for estimation from observational or trial data (e.g. Moodie et al. [12]; Almirall et al. [2]; Henderson et al. [8]; Zhang et al. [20]; Zhao et al. [21, 22]). Some of these rely on knowledge or assumptions on the process by which decisions on treatment *A* are reached, which is straightforward for a randomised trial, and some of which rely on modelling the evolution of the states *S* as time proceeds. A particular case of the former is the g-estimation procedure proposed by Robins [17], and beautifully summarised by Moodie et al. [12]. A special case of the latter is the so-called regret-regression approach that was proposed independently by Almirall et al. [2] and Henderson et al. [8]. These methods all formulate the problem in terms of the structural nested mean models (SNMMs) described by Robins [17]. An alternative approach based on marginal structural models has been proposed by Orellana et al. [16], which allows the estimation of simple dynamic treatment rules. For example, the decision when to start a treatment may be based on state measurements progressing beyond a threshold, which must be determined. We will focus on the SNMM approaches in this paper.

An estimation method is doubly robust if it gives consistent parameter estimates whenever *either* the state mechanism *S* *or* the action process *A* has been modelled correctly. The g-estimation method is founded, as stated, on knowledge of the decision or action process *A*. If there is also assumed knowledge of the state *S* mechanism then a doubly robust form can be constructed (Robins [17]). It is of interest therefore to ask whether a doubly robust form of the regret-regression approach can be found. In Sect. 3 below we propose such a modification and we show how it is closely linked to doubly robust g-estimation. In Sect. 4 we use simulation to compare performance of various methods in terms of efficiency and robustness, and in Sect. 5 we illustrate use in treatment of patients on long term anticoagulation therapy.

## Modelling Dynamic Treatment Regimes

We assume that we have data from *n* independent individuals, each observed according to the same visit schedule consisting of *K* visits. At visit *j*, measurements are taken which define the current state *S*_*j*_ of the patient and a treatment decision *A*_*j*_ is made. After *K* visits an outcome *Y* is measured. Our aim is to use the observed data to determine the optimal dynamic treatment regime to maximise the outcome *Y*. As an illustration we will use data from a study investigating patients taking the anticoagulation treatment warfarin to avoid abnormal blood clotting. Here measurements of blood-clotting potential are taken at each visit, defining *S*_*j*_, and a dose of warfarin is prescribed, defining the action *A*_*j*_. The final outcome *Y* is the time spent with blood-clotting time within a target range over the entire course of follow-up.

Taking a potential outcomes (or counterfactual) approach (see for example Greenland et al. [7]), let  be the set of all possible actions that could be taken at visit *j*, and let  be the set of all possible treatment regimes up to visit *j*. For , $\bar{S}_{j}(\bar{a}_{j-1}) = (S_{1}, S_{2}(a_{1}),\ldots ,S_{j}(\bar{a}_{j-1}))$ denotes the potential state history under the treatment regime $\bar{a}_{j-1}$. Similarly, $Y(\bar{a}_{K})$ denotes the potential outcome under the treatment regime .

We make the consistency assumption that the observed state history $\bar{S}_{K} = (S_{1}, \ldots, S_{K})$ is equal to the potential state history $\bar{S}(\bar{a}_{K-1})$ under the observed treatment regime $\bar{a}_{K} = \bar{A}_{K}=(A_{1},\ldots,A_{K})$ and that the observed outcome *Y* is equal to the potential outcome $Y(\bar{a}_{K})$ under the observed treatment regime $\bar{a}_{K} = \bar{A}_{K}$. In short, this means that the method by which treatments are assigned does not affect the values of the future states or the outcome (see Cole and Frangakis [5], for a thorough discussion of the consistency assumption). Throughout this paper we will therefore replace potential outcomes notation, e.g. $E(Y|\bar{S}(a_{K-1}),\bar{a}_{K})$ for the expected value of the potential outcome $Y(\bar{a}_{K})$ conditional on the treatment regime $\bar{a}_{K}$ and potential state history $\bar{S}(\bar {a}_{K-1})$, with the observed outcomes notation $E(Y|\bar{S}_{K},\bar{A}_{K})$.

We also make the assumption of no unmeasured confounders, which means that the choice of treatment to be received does not depend on potential future states or the potential outcome except through observed state and treatment history. When no drop-out occurs this assumption is equivalent to exchangeability. It enables us to estimate causal effects from observational data (see Hernán and Robins [11], for a discussion of the exchangeability assumption). We make a third assumption of positivity, that the optimal treatment regime has a positive probability of being observed in the data or, in the case of a continuous treatment, that it is identifiable from the observed data (see Cole and Hernán [4], for a discussion of positivity and Henderson et al. [8], for the extension in the continuous case). All three assumptions are standard in causal inference.

Let $\bar{S}_{j}=(S_{1},\ldots,S_{j})$ be the observed measurement history up to and including visit *j*, and $\bar{A}_{j}=(A_{1},\ldots,A_{j})$ be the history of actions taken up to visit *j*. A dynamic treatment regime *d* is defined by a set of decision rules, $d = (d_{1}(S_{1}), \ldots, d_{j}(\bar{S}_{j}, \bar{A}_{j-1}), \ldots, d_{K}(\bar{S}_{K},\bar {A}_{K-1}))$, which prescribe an action to be taken at each visit given all information available at the time of the visit, including the current state *S*_*j*_. The optimal dynamic treatment regime $d^{\operatorname{opt}}$ is the one which optimises the expected value of the outcome *Y*.

A naive approach to modelling the outcome would be to regress *Y* on state history $\bar{S}_{K}$ and action history $\bar{A}_{K}$. However, this ignores the potential effect of previous actions $\bar{A}_{j-1}$ and states $\bar{S}_{j-1}$ on the current state $\bar{S_{j}}$. Including the state *S*_*j*_ in the analysis may introduce bias because action history $\bar{A}_{j-1}$ and state history $\bar{S}_{j-1}$ may influence both the current state *S*_*j*_ and the outcome *Y*.

This problem can be solved by modelling quantities which isolate the causal effect of treatment *A*_*j*_ on *Y* (see Hernán [9], for a discussion of the use of causal effects in causal inference). Murphy [15] proposed the use of regret functions which measure the expected decrease in *Y* due to an action *a*_*j*_ taken at time *j* compared to the optimal action, given that optimal actions are used in the future. The regret at time *j* is defined by

1 $$\begin{aligned} \mu_j(a_j | \bar{S_j}, \bar{A}_{j-1}) &= E\bigl(Y\bigl(a_1,\ldots ,a_{j-1},d^{\operatorname{opt}}_j,\ldots,d^{\operatorname{opt}}_K \bigr)\big|\bar{S}_j,\bar{a}_{j-1}=\bar {A}_{j-1}\bigr) \\ &\quad - E\bigl(Y\bigl(a_1,\ldots,a_j,d^{\operatorname{opt}}_{j+1}, \ldots,d^{\operatorname{opt}}_K\bigr)\big|\bar{S}_j,\bar {a}_{j-1}=\bar{A}_{j-1}\bigr). \end{aligned}$$

As an alternative Robins [17] suggested using a blip function which compares actions to a reference action *a* ^0^. The blip measures the expected change in *Y* when action *a*_*j*_ is taken at time *j* compared to *a* ^0^, assuming future actions are *a* ^0^,

2 $$\begin{aligned} \gamma_j(a_j | \bar{S_j}, \bar{A}_{j-1}) &= E\bigl(Y\bigl(a_1,\ldots ,a_{j-1},d^{0}_j,\ldots,d^{0}_K \bigr)\big|\bar{S}_j,\bar{a}_{j-1}=\bar{A}_{j-1}\bigr) \\ &\quad - E\bigl(Y\bigl(a_1,\ldots,a_j,d^{0}_{j+1}, \ldots,d^{0}_K\bigr)\big|\bar{S}_j,\bar {a}_{j-1}=\bar{A}_{j-1}\bigr), \end{aligned}$$

where the reference regime *d* ^0^ specifies that all actions are set to *a* ^0^.

It has been argued by Robins [17] that correct models can be specified more easily for blip functions because a comparison to a reference regime can be envisaged more readily by clinicians than a comparison to an unspecified optimal regime. However, determining the optimal regime from models for the blip functions can be computationally challenging, whereas the optimal action $a_{j}^{\operatorname{opt}}$ immediately follows from the form of the regret function because by construction $\mu_{j}(a_{j}^{\operatorname{opt}}|\bar{S_{j}}, \bar{A}_{j-1})=0$. Also, because the form of the optimal treatment immediately follows from the form of the regret function, the use of regrets enables us to restrict our attention to decision rules with simple forms (see also Rosthøj et al. [19]). For these reasons we will use regret functions in the rest of this paper.

## Estimating Optimal Dynamic Treatment Regimes

Two methods which can be used to estimate the optimal dynamic treatment regime are g-estimation (Robins [17], see also Moodie et al. [12]) and regret-regression, which was proposed independently by Henderson et al. [8] and Almirall et al. [2].

### G-estimation

In order to estimate an optimal dynamic treatment regime using g-estimation, we must first specify models for the regret functions $\mu _{j}(a_{j} | \bar{S_{j}}, \bar{A}_{j-1})$. The form of the regret functions determines the form of the optimal treatment rules. Hereafter, when we refer to an optimal decision rule we therefore mean the decision rule of the specified form which optimises the expected outcome. Models for *μ*_*j*_ may depend on parameters *ψ*, which may be shared across time-points. See Moodie et al. [12] and Moodie et al. [13] for examples of models with different parameters at different time-points. We then define

$$H_j = H_j(\psi) = Y + \sum _{k\geq j} \mu_k(A_k | \bar{A}_{k-1},\bar {S}_k;\psi), $$

which provides an estimate of the expected outcome in the counterfactual event that optimal decisions are followed from time *j* onwards (Robins [17]; Moodie et al. [12]). For conciseness we shorten $\mu_{j}(A_{j} | \bar{A}_{j-1},\bar{S}_{j};\psi)$ to *μ*_*j*_ for the remainder of this paper.

We also specify models for the probability density $f(a_{j}|\bar{S}_{j},\bar {A}_{j-1})$ for the assigned value of the action *A*_*j*_, conditional on state and action history, and for $E(H_{j}|\bar{S}_{j},\bar{A}_{j-1})$. We can then form the g-estimation equations

3 $$\begin{aligned} EE^{\mathit{GE}} (\psi) & = \sum_{j=1}^K \bigl( H_{j} - E(H_{j}|\bar{S}_{j},\bar {A}_{j-1}) \bigr) \bigl( g_j(A_{j}| \bar{S}_j,\bar{A}_{j-1}) \\ &\quad - E_{A_{j}} \bigl(g_j(A_{j}| \bar{S}_j,\bar{A}_{j-1}) \bigr) \bigr) \end{aligned}$$

for some functions $g_{j}(A_{j}|\bar{S}_{j},\bar{A}_{j-1})$ of the same dimension as *ψ*. It has been shown that solutions $\hat {\psi}^{\mathit{GE}}$ to *E*(*EE* ^*GE*^(*ψ*))=0 provide consistent estimates of *ψ* if the regret functions are correctly modelled and either the model specified for $f(a_{j}|\bar{S}_{j},\bar{A}_{j-1})$ or the model specified for $E(H_{j}|\bar{S}_{j},\bar{A}_{j-1})$ is correct (Robins [17]). We give a simpler proof in Appendix A.1. G-estimation is therefore doubly robust in the sense discussed in the Introduction.

A simple choice for the functions *g*_*j*_(*A*_*j*_) is (Moodie et al. [12]):

$$g^{\operatorname{simp}}_j(A_j | \bar{S}_j, \bar{A}_{j-1}) = E \biggl( \frac{\partial\mu _j}{\partial\psi} \bigg| \bar{S}_j, \bar{A}_j \biggr), $$

which can be calculated easily from the *μ*_*j*_(*ψ*). The alternative

4 $$\begin{aligned} g^{\operatorname{eff}}_j(A_j | \bar{S}_j, \bar{A}_{j-1}) = & E \biggl( \frac{\partial H_j}{\partial\psi} \bigg| \bar{S}_j, \bar{A}_j \biggr) \\ = & E \biggl( \sum_{k\geq j} \frac{\partial\mu_k}{\partial\psi} \bigg| \bar{S}_j, \bar{A}_j \biggr) \end{aligned}$$

gives Robins’ [17] locally efficient semiparametric estimator of *ψ*. While $g^{\operatorname{eff}}$ has been shown to be more efficient than $g^{\operatorname{simp}}$ (Robins [17]), it can be more complicated to calculate because it requires expected values of *μ*_*k*_ conditional on $(\bar{S}_{j},\bar {A}_{j})$ for *k*>*j*. In turn these require conditional expectations of (functions of) all *S*_*k*_ and *A*_*k*_ for *k*>*j* and hence detailed knowledge of both state and action evolution processes.

### Regret-regression

Murphy [15] showed that $E(Y|\bar{S}_{K}, \bar{A}_{K})$ can be decomposed into a sum of regret functions *μ*_*j*_ and nuisance functions $\phi _{j}(S_{j}|\bar{S}_{j-1}, \bar{A}_{j-1})$ as follows:

5 $$ E(Y|\bar{S}_K,\bar{A}_{K}) = \beta_0 + \sum _{j=1}^K \phi_j (S_j|\bar {S}_{j-1}, \bar{A}_{j-1}) - \sum _{j=1}^K\mu_j( A_j | \bar{S}_j,\bar{A}_{j-1}). $$

The nuisance function $\phi_{j} (S_{j}|\bar{S}_{j-1}, \bar{A}_{j-1})$ for *j*≥2 is defined to be

6 $$\begin{aligned} \phi_j (S_j|\bar{S}_{j-1}, \bar{A}_{j-1}) &= E\bigl(Y\bigl(a_1,\ldots ,a_{j-1},d^{\operatorname{opt}}_j,\ldots,d^{\operatorname{opt}}_K \bigr)\big|\bar{S}_j,\bar{A}_{j-1}\bigr) \end{aligned}$$

7 $$\begin{aligned} &\quad - E\bigl(Y\bigl(a_1,\ldots,a_{j-1},d^{\operatorname{opt}}_j, \ldots,d^{\operatorname{opt}}_K\bigr)\big|\bar {S}_{j-1}, \bar{A}_{j-1}\bigr), \end{aligned}$$

with $\phi_{1}(S_{1}) = E(Y(d^{\operatorname{opt}}_{1},\ldots,d^{\operatorname{opt}}_{K})|S_{1}) - E(Y(d^{\operatorname{opt}}_{1},\ldots,d^{\operatorname{opt}}_{K}))$. The function $\phi_{j}(S_{j}|\bar {S}_{j-1}, \bar{A}_{j-1})$ expresses the change in the expected value of *Y* due to the measurement of *S*_*j*_ when optimal decision rules are used in the future. Note that $E_{S_{j}}(\phi_{j}(S_{j}|\bar{S}_{j-1}, \bar {A}_{j-1})) = 0$ follows from the definition of $\phi_{j}(S_{j}|\bar {S}_{j-1}, \bar{A}_{j-1})$. Note also that the decomposition (5) *requires* the nuisance and regret functions to be defined as differences of expectations under the assumption that optimal policies are followed at future time-points. There is no similar decomposition with non-negative {*μ*_*j*_} based on a comparison with non-optimal policies, such as the blip functions suggested by Robins [17] (see Appendix B).

The decomposition (5) can be used to estimate regret parameters *ψ* if models are specified for the $\phi_{j}(S_{j}|\bar {S}_{j-1}, \bar{A}_{j-1})$ (Henderson et al. [8]; Almirall et al. [2]). To satisfy the condition $E_{S_{j}}(\phi_{j}(S_{j}|\bar{S}_{j-1}, \bar {A}_{j-1})) = 0$, Henderson et al. [8] suggested the form

$$\phi_j (S_j|\bar{S}_{j-1}, \bar{A}_{j-1}) = \beta_{j}^T(\bar {S}_{j-1},\bar{A}_{j-1}) \bigl(S_{j} - E(S_j|\bar{S}_{j-1},\bar{A}_{j-1})\bigr), $$

where $\beta_{j}^{T}(\bar{S}_{j-1},\bar{A}_{j-1})$ is a coefficient which may depend on the state and action history before time *j*. Under this approach a model must be specified for $E(S_{j}|\bar{S}_{j-1},\bar {A}_{j-1})$. Parameters can be estimated using least squares, which is equivalent to solving *E*(*EE* ^*RR*^(*ψ*))=0, where *EE* ^*RR*^(*ψ*) are the regret-regression estimating equations

8 $$ EE^{\mathit{RR}}(\psi) = \bigl(Y - E(Y|\bar{S}_{K}, \bar{A}_{K})\bigr) \sum_j \frac{\partial \mu_{j}}{\partial\psi}. $$

A proof is given in Appendix A.2 that the resulting estimates $\hat{\psi}^{\mathit{RR}}$ are consistent estimates for *ψ* provided the regret functions $\mu_{j}(a_{j} | \bar{S_{j}}, \bar {A}_{j-1})$ and the nuisance functions $\phi_{j}(S_{j}|\bar{S}_{j-1}, \bar {A}_{j-1})$ have been modelled correctly.

A natural question to ask is whether we can formulate a doubly robust version of regret-regression, which is robust to misspecification of either $\phi_{j}(S_{j}|\bar{S}_{j-1}, \bar{A}_{j-1})$ or the probability density $f(a_{j}|\bar{S}_{j},\bar{A}_{j-1})$ of assigning action *A*_*j*_. A naive extension of the estimating equations (8) would be

9 $$ EE^{\operatorname{naive}}(\psi) = \bigl(Y - E(Y|\bar{S}_{K}, \bar{A}_{K})\bigr) \sum_j \biggl( \frac {\partial\mu_{j}}{\partial\psi} - E_{A_j} \biggl(\frac{\partial\mu_{j}}{\partial\psi} \biggr) \biggr). $$

However, the resulting estimates $\hat{\psi}^{\operatorname{naive}}$ are not consistent if the $\phi_{j}(S_{j}|\bar{S}_{j-1}, \bar{A}_{j-1})$ are misspecified because when we take the expectation over *Y* the left bracket of (9) retains some dependence on *A*_*j*_ for *j*=1,…,*K*−1 (see Appendix A). However, we obtain consistent estimates with the double-robustness property if we replace the sum in (9) with the contribution just from the final term:

10 $$ EE^{\mathit{DRRR}}(\psi) = \bigl(Y - E(Y|\bar{S}_{K}, \bar{A}_{K})\bigr) \biggl(\frac {\partial\mu_{K}}{\partial\psi} - E_{A_K} \biggl( \frac{\partial\mu_{K}}{\partial\psi} \biggr) \biggr). $$

The estimators $\hat{\psi}^{\mathit{DRRR}}$ derived from (10) will be consistent because *E*(*EE* ^*DRRR*^)=0.

Note that  So the doubly robust regret-regression estimating equations (10) are identical to the final (*j*=*K*) term of the g-estimating equations (3) with $g_{j} = g^{\operatorname{simp}}_{j}$ when $E(Y|\bar{S}_{K},\bar{A}_{K})$ is modelled in the same way. Specification of $E(H_{j}(\psi)|\bar {S}_{j},\bar{A}_{j-1})$ is equivalent to specification of the nuisance functions $\phi_{j}(S_{j}|\bar{S}_{j-1}, \bar{A}_{j-1})$ for regret-regression because

$$E\bigl(H_j(\psi)|\bar{S}_j,\bar{A}_{j-1} \bigr) = \beta_0+ \sum_{k=1}^{j} \phi_k (S_k|\bar{S}_{k-1}, \bar{A}_{k-1}) - \sum_{k=1}^{j-1} \mu_k( A_k | \bar{S}_k,\bar{A}_{k-1};\psi), $$

(see Appendix A.1). It may be difficult to identify an appropriate model for either $E(H_{j}(\psi)|\bar{S}_{j},\bar {A}_{j-1})$ or $\phi_{j}(S_{j}|\bar{S}_{j-1}, \bar{A}_{j-1})$, and the choice of which to specify is likely to depend on the context. See Henderson et al. [8] and Rosthøj et al. [19] for further discussion about modelling $\phi_{j}(S_{j}|\bar{S}_{j-1}, \bar{A}_{j-1})$. We recommend taking the models to be as general as possible, see Sect. 5 for an example. Since these models are not of direct interest, it is safer to err on the side of overfitting (Henderson et al. [8]). We will show via simulation studies in Sect. 4 that restricting to the final term in this way results in a loss of precision for $\hat{\psi}^{\mathit{DRRR}}$ compared to $\hat {\psi}^{\mathit{GE}}$.

## Simulation

We demonstrate the behaviour of $\hat{\psi}^{\mathit{GE}}$ with $g_{j}=g_{j}^{\operatorname{simp}}$, $\hat{\psi}^{\mathit{GE}}$ with $g_{j}=g_{j}^{\operatorname{eff}}$, $\hat{\psi }^{\mathit{RR}}$ and $\hat{\psi}^{\mathit{DRRR}}$ using a simulation study. We generated data from 1000 patients, followed-up over 5 time-points. States were normally distributed with *E*(*S*_1_)=0.5, *E*(*S*_*j*_|*A*_*j*−1_)=(0.5−*A*_*j*−1_) for *j*>1 and residual variance $\sigma^{2}_{s}=1$. Actions were generated as *A*_*j*_∼*U*(1.25,3) when *S*_1_>0.5 and *A*_*j*_∼*U*(0,1.75) when *S*_1_≤0.5. By definition *μ*_*j*_ is non-negative, so regret functions were taken to be quadratic with $\mu _{j}(a_{j}|\bar{S}_{j},\bar{A}_{j-1}) = \psi_{1} (a_{j} - \psi_{2} S_{j})^{2}$, with *ψ*_1_=6 and *ψ*_2_=2. The optimal action at visit *j*, $a_{j}^{\operatorname{opt}}$, is the action satisfying $\mu_{j}(a_{j}^{\operatorname{opt}}|\bar{S}_{j},\bar {A}_{j-1})=0$, giving $a_{j}^{\operatorname{opt}}= \psi_{2} S_{j}$. Note that the optimal action may be negative, even though the observed actions are always positive. In practice this would mean that estimated optimal actions had been extrapolated to a region of  that had not been observed in the data. Such an extrapolation would only be appropriate if regret functions had been modelled correctly. Outcomes *Y* were normally distributed with

$$E(Y|\bar{S}_K,\bar{A}_K) = 30 - 5 \bigl(S_1 - E(S_1)\bigr) - \sum_{j=2}^5 (5 + 2 A_{j-1}) \bigl(S_j - E(S_j|A_{j-1}) \bigr) - \sum_{j=1}^{5} \mu_j $$

and variance $\sigma_{y}^{2}=1$.

For both g-estimation and regret-regression, parameters were estimated using a two-stage process. In the first stage the model for the state distribution was fitted to the observed states and, if required, the model for assigning actions was fitted to the observed actions. For regret-regression these models were then used to estimate the residuals $Y-E(Y|\bar{S}_{K},\bar{A}_{K})$ using the decomposition (5), and parameters estimated using least squares. For all other methods the models were used to determine the corresponding estimating equations, which were solved numerically. Standard errors were calculated using bootstrapping with 100 bootstrap samples.

Parameter estimates $\hat{\psi}$ were obtained using correctly and incorrectly specified models for *S*_*j*_ and *A*_*j*_. The misspecified model for *S*_*j*_ assumed *S*_*j*_∼*N*(0.5,1), and so ignored the dependence of the states on the previous action. In the misspecified action model the actions were assumed to be uniformly distributed between 0 and 3.

Table 1 shows results for *ψ*_2_, which is the parameter of most interest since it determines the optimal dose. Results for other parameters are not reported, but lead to similar conclusions. Coverage probability is estimated by the proportion of simulations for which the estimated confidence interval contains the true parameter value. Parameter estimates were discarded when convergence was not achieved.

**Table 1:** Simulation results for *ψ*_2_ using regret-regression (RR), doubly-robust regret-regression (DRRR), g-estimation with $g=g^{\operatorname{simp}}$ (GE SIMP) and g-estimation with $g=g^{\operatorname{eff}}$ (GE EFF). Reported are means of parameter estimates with standard deviation of parameter estimates in brackets, means of estimated standard errors, coverage probability, root-mean-square error and the number of simulated data sets for which convergence was achieved. Results are based on 1000 samples of size *n*=1000

Misspecification	Method	Mean (SD)	Mean of SE estimates	Coverage	RMSE	Number converged
No misspecification	RR	2.000 (0.001)	0.001	0.938	0.001	975
	DRRR	2.000 (0.038)	0.042	0.972	0.038	1000
	GE SIMP	2.000 (0.027)	0.040	0.998	0.027	1000
	GE EFF	2.000 (0.008)	0.008	0.973	0.008	988
Misspecified *P*(*S*_*j*_)	RR	1.957 (0.003)	0.003	0	0.043	994
	DRRR	1.994 (0.128)	0.145	0.991	0.128	999
	GE SIMP	1.987 (0.072)	0.090	0.993	0.073	1000
	GE EFF	1.987 (0.072)	0.091	0.994	0.073	1000
Misspecified *P*(*A*_*j*_)	RR	2.000 (0.001)	0.001	0.933	0.001	972
	DRRR	2.007 (0.058)	0.065	0.986	0.059	970
	GE SIMP	1.999 (0.028)	0.034	0.993	0.028	989
	GE EFF	2.000 (0.008)	0.008	0.958	0.008	942
Misspecified *P*(*S*_*j*_) and *P*(*A*_*j*_)	RR	1.957 (0.003)	0.003	0	0.043	797
	DRRR	1.840 (0.233)	0.220	0.814	0.283	800
	GE SIMP	1.960 (0.044)	0.057	0.990	0.059	799
	GE EFF	1.960 (0.044)	0.057	0.980	0.059	799

When models for both *S*_*j*_ and *A*_*j*_ were specified correctly, parameter estimates were consistent using all estimation methods. The most efficient method was RR. For GE SIMP estimated standard errors tended to be too high, leading to over-coverage of confidence intervals.

When the model for *S*_*j*_ is misspecified, RR results are slightly biased, with none of the estimated confidence intervals containing the true parameter value *ψ*_2_=2. All other methods are robust to misspecification of the state model, and gave consistent parameter estimates. The GE EFF estimating equations for this scenario are identical to the GE SIMP estimating equations because the incorrect model for *S*_*j*_ has been used when calculating expressions for the $g_{j}^{\operatorname{eff}}$; because the misspecified model for *S*_*j*_ is independent of *A*_*j*−1_, only the term involving *μ*_*j*_ in (4) depends on *A*_*j*_, and all other terms therefore cancel when subtracting $E_{A_{j}} (g_{j}(A_{j}|\bar{S}_{j},\bar {A}_{j-1}))$ from $g_{j}(A_{j}|\bar{S}_{j},\bar{A}_{j-1})$. The DRRR method was less efficient than GE SIMP and GE EFF. For all the methods overestimation of standard errors gave over-coverage of confidence intervals.

When the model for *A*_*j*_ is misspecified, all methods give consistent parameter estimates. For RR this is because the method does not depend on the model for *A*_*j*_, and all other methods are robust to misspecification of the action model. Again, the most efficient method is RR.

When models for both *S*_*j*_ and *A*_*j*_ are misspecified, none of the methods would be expected to give consistent parameter estimates. Here all methods gave biased results, with DRRR parameter estimates being the most biased. RR has the smallest root mean squared error, with similar bias but smaller standard errors compared to GE SIMP. Parameter estimates from misspecified models took longer to converge, as indicated by the low convergence rates.

The bias caused by misspecification of state and action models in our simulation study was smaller than might be expected from a previous simulation study (Almirall et al. [2]). This could be because we have focussed on a continuous treatment decision, whereas Almirall et al. considered only binary actions. Model misspecification in the Almirall et al. study was generated by multiplying estimated state values by random noise of varying amplitudes. In contrast, our simulation study aimed to explore model misspecifications that might occur in practice, such as omitting variables from the state and action models.

## Example: Blood-Clotting

We illustrate the methods with data taken from 303 patients at risk of thrombosis who were receiving long-term anticoagulation therapy for abnormal blood-clotting. These data have been analysed previously by Rosthøj et al. [18] and by Henderson et al. [8]. The ability of the blood to clot was measured using the International Normalised Ratio (INR), with high values indicating that the blood clots too slowly, increasing the risk of haemorrhage, and low values indicating fast clotting-times with an increased risk of thrombosis. Each patient attended 14 clinic visits at which their INR was measured and their dose of anticoagulant was adjusted accordingly. The aim of therapy is to maintain a patient’s INR within a target range, which is pre-specified for each patient.

As an outcome for analysis we used the proportion of time over follow-up that was spent with the INR within target range. The final dose adjustment did not contribute to the outcome, and we treated the first four clinic visits as a stabilisation period, giving *K*=9. States *S*_*j*_ are defined to be the standardised difference between the INR at the *j*th visit and the target range. Actions *A*_*j*_ are defined to be the change in anticoagulant dose at the *j*th visit. With these definitions *S*_*j*_=0 for 50 % of state observations and *A*_*j*_=0 for 60 % of actions taken.

We modelled the regrets as quadratic functions, depending on the previous two states and the previous action:

$$\mu_j (a_j | \bar{S}_j, \bar{A}_{j-1}; \psi) = \psi_{1} (a_j - \psi_{2}S_j - \psi_{3}S_{j-1} - \psi_4 A_{j-1})^2. $$

To model the states we used a mixture model with logistic and normal components to account for the high number of zero states. Linear predictors for both models were allowed to depend on the previous four states and actions, as well as a number of interactions between them. The model for the actions was defined in the same way.

Parameters were estimated using RR, DRRR and GE SIMP, with standard errors by bootstrap with 1000 resamplings. We were unable to implement the more efficient method GE EFF because of the extra complexity introduced by the dependence of the regret functions on the previous state and the previous action. In this case no terms in $g_{j}(A_{j}) - E_{A_{j}} (g_{j}(A_{j}) | \bar{S}_{j}, \bar{A}_{j})$ automatically cancelled, as was the case for the simulation study. So, for example, it would be necessary to calculate *E*(*∂μ*_9_/*∂ψ*|*S*_1_,*A*_1_) by integrating out all other *S*_*j*_ and *A*_*j*_. In this complicated scenario we found such calculations to be algebraically intractable.

Results are given in Table 2. Parameter estimates from RR, DRRR and GE SIMP are similar, although the RR results tend to favour slightly more extreme changes of dose than the GE SIMP results. The difference between RR and GE SIMP results could indicate some model misspecification, but standard errors are too large to draw any firm conclusions. The DRRR standard errors were substantially larger than the GE SIMP standard errors. We can therefore place most confidence in the GE SIMP parameter estimates because GE SIMP is the most efficient estimation method with the double-robustness property. Some bootstrap samples (3 out of 1000) did not converge using RR, and for others there was a tendency for *ψ*_1_ to be estimated close to 0. This could explain the larger standard errors estimated for RR compared to GE SIMP.

**Table 2:** Results for the blood-clotting example using regret-regression (RR), doubly robust regret-regression (DRRR) and g-estimation with $g=g^{\operatorname{simp}}$ (GE SIMP). Reported are estimated parameter values with standard errors in brackets

Parameter	RR	DRRR	GE SIMP
*ψ*_1_	0.093 (0.065)	0.099 (0.059)	0.113 (0.046)
*ψ*_2_	−2.477 (1.594)	−2.264 (1.856)	−2.267 (0.435)
*ψ*_3_	−1.729 (0.976)	−1.817 (0.966)	−1.535 (0.683)
*ψ*_4_	−0.993 (0.517)	−1.058 (0.608)	−0.822 (0.339)

The estimates for *ψ*_2_ indicate that the dose should be increased if the current state is too low and should be decreased if it is too high, as would be expected. Negative values of *ψ*_3_ indicate that if the previous state is below range then the current dose should be adjusted upwards, and if it is above range then the current dose should be adjusted down. Similarly, estimates for *ψ*_4_ indicate that if the previous dose was increased then the current dose should be reduced and vice versa. So, for example, a patient whose current INR measurement is *S*_*j*_=0.5, and who previously also had high INR, *S*_*j*−1_=0.5, and whose dose was reduced, *A*_*j*−1_=−0.5, would be recommended to reduce their dose by 1.44 according to the GE SIMP estimates. By comparison, a patient who also had *S*_*j*_=0.5, but whose INR was previously too low, *S*_*j*−1_=−0.5, resulting in an increase of dose *A*_*j*−1_=0.5, would be recommended to reduce their dose by a smaller amount of 0.80.

In summary, both methods give plausible parameter estimates, but RR standard errors seem large in comparison with GE SIMP standard errors. The simulation results suggest that standard errors estimated using GE SIMP could also be overly conservative.

## Discussion

We have demonstrated that two methods which have been proposed for estimating optimal dynamic treatment regimes, regret-regression and g-estimation, are closely related. Formulating a doubly robust version of regret-regression led to a truncated version of the g-estimation equations.

The regret-regression approach is efficient when the model for states *S*_*j*_ is correctly specified. No model for actions *A*_*j*_ is required. G-estimation, on the other hand, can be applied when the action model is known, without the need to model states correctly. This is perhaps the best approach for trial data, where actions are randomised and hence fully understood. For observational data it may be the case that the natural process of state evolution is easier to model than the subjective actions chosen by health personnel. G-estimation is doubly robust in the sense that parameter estimates are consistent provided that either the states or the actions are modelled correctly. An assumption of no unmeasured confounders is necessary for inference in both cases.

Regret-regression outperforms efficient g-estimation even when the latter makes use of correct specification of *both* action and state models. However, it performs poorly when the state model is misspecified, whereas efficient g-estimation is robust. Given that the states are fully observed one can argue that careful attention to modelling and diagnostics should reduce or remove the risk of major misspecification. Nonetheless our recommendation is to attempt efficient g-estimation whenever possible. Unfortunately, as in the blood clotting application, when the regret and state models are fairly complex it can be difficult or in practice impossible to obtain the functions *H*_*j*_(*ψ*) defined at (1) that are required for implementation.

Biases resulting from model misspecification were smaller than might have been expected from a previous simulation study (Almirall et al. [2]). One difference here is that we have focussed on continuous rather than binary treatment decisions. It would be interesting to see if such small biases persist for other forms of regret functions and more complicated models. We have assumed throughout that regret functions have been specified correctly. We leave investigation of the effects of regret misspecification for future work.

## Appendix A: Consistency of Estimating Equations

### A.1 Consistency of $\hat{\psi}^{\mathit{GE}}$

We assume throughout that regret functions have been modelled correctly. We will show that *E*(*EE* ^*GE*^(*ψ*))=0 providing that either the states or the treatment probabilities have also been modelled correctly, where

$$\begin{aligned} EE^{\mathit{GE}}(\psi) &= \sum_{j=1}^K \bigl( H_{j}(\psi) {-} E\bigl(H_{j}(\psi)|\bar {S}_{j},\bar{A}_{j-1}\bigr) \bigr)\\&\quad\times \bigl( g_j(A_{j}|\bar{S}_j,\bar{A}_{j-1}) {-} E_{A_{j}} \bigl(g_j(A_{j}|\bar {S}_j,\bar{A}_{j-1}) \bigr) \bigr). \end{aligned}$$

Consider

$$\begin{aligned} &E\bigl(H_j(\psi)|\bar{S}_j,\bar{A}_{j-1} \bigr) = E_{A_j,S_{j+1},\ldots,S_K,A_K,Y} \biggl(Y {+} \sum_{k\geq j} \mu_k(A_k | \bar{S}_k,\bar{A}_{k-1}; \psi) \vert \bar{S}_j,\bar{A}_{j-1} \biggr). \end{aligned}$$

Taking the expectation over *Y*, and using the decomposition (5), we get

$$\begin{aligned} E\bigl(H_j(\psi)|\bar{S}_j,\bar{A}_{j-1} \bigr) = & E_{A_j,S_{j+1},\ldots ,S_K,A_K} \Biggl(\beta_0+ \sum _{k=1}^K \phi_k (S_k| \bar{S}_{k-1}, \bar{A}_{k-1}) \\ & - \sum_{k=1}^{j-1}\mu_k( A_k | \bar{S}_k,\bar{A}_{k-1};\psi) \vert \bar{S}_j,\bar{A}_{j-1} \Biggr). \end{aligned}$$

None of the terms on the right-hand side depend on *A*_*K*_, so

$$\begin{aligned} E\bigl(H_j(\psi)|\bar{S}_j,\bar{A}_{j-1} \bigr) = & E_{A_j,S_{j+1},\ldots,S_K} \Biggl(\beta_0+ \sum _{k=1}^K \phi_k (S_k| \bar{S}_{k-1}, \bar{A}_{k-1}) \\ & - \sum_{k=1}^{j-1} \mu_k( A_k | \bar{S}_k,\bar{A}_{k-1};\psi) \vert \bar{S_j},\bar{A}_{j-1} \Biggr). \end{aligned}$$

Now the only term depending on *S*_*K*_ is $\phi_{K} (S_{K}|\bar{S}_{K-1}, \bar {A}_{K-1})$, which has expectation zero, so

$$\begin{aligned} E\bigl(H_j(\psi)|\bar{S}_j,\bar{A}_{j-1} \bigr) = & E_{A_j,S_{j+1},\ldots ,S_{K-1},A_{K-1}} \Biggl(\beta_0+ \sum _{k=1}^{K-1} \phi_k (S_k|\bar {S}_{k-1}, \bar{A}_{k-1}) \\ & - \sum_{k=1}^{j-1}\mu_k( A_k | \bar{S}_k,\bar{A}_{k-1};\psi) \vert \bar{S}_j,\bar{A}_{j-1} \Biggr). \end{aligned}$$

Repeating these steps to take expectations of *A*_*K*−1_, *S*_*K*−1_, …, *A*_*j*_, we get

$$E\bigl(H_j(\psi)|\bar{S}_j,\bar{A}_{j-1} \bigr) = \beta_0+ \sum_{k=1}^{j} \phi_k (S_k|\bar{S}_{k-1}, \bar{A}_{k-1}) - \sum_{k=1}^{j-1} \mu_k( A_k | \bar{S}_k,\bar{A}_{k-1};\psi). $$

Let $\tilde{\phi}_{j}(S_{j}|\bar{S}_{j-1}, \bar{A}_{j-1})$ be the postulated model for $\phi_{j}(S_{j}|\bar{S}_{j-1},\bar{A}_{j-1})$, so $E(H_{j}(\psi)|\bar{S}_{j},\bar{A}_{j-1})$ is modelled as $\tilde{\beta}_{0}+ \sum_{k=1}^{j} \tilde{\phi}_{k} (S_{k}|\bar{S}_{k-1}, \bar{A}_{k-1}) - \sum_{k=1}^{j-1}\mu_{k}( A_{k} | \bar{S}_{k},\bar{A}_{k-1};\psi)$. Then

$$\begin{aligned} EE^{\mathit{GE}}(\psi) = & \sum_{j=1}^K \Biggl( Y + \sum_{k=1}^{K} \mu_k( A_k | \bar{S}_k,\bar{A}_{k-1};\psi) - \tilde{\beta}_0 - \sum _{k=1}^{j} \tilde {\phi}_k (S_k|\bar{S}_{k-1}, \bar{A}_{k-1}) \Biggr) \\ & \times \bigl( g_j(A_{j}| \bar{S}_j,\bar{A}_{j-1}) - E_{A_{j}} \bigl(g_j(A_{j}|\bar{S}_j, \bar{A}_{j-1}) \bigr) \bigr). \end{aligned}$$

The expectation of the estimating equations over all random variables is then

$$\begin{aligned} & E_{\bar{S}_K,\bar{A}_K,Y}\bigl(EE^{\mathit{GE}}(\psi)\bigr) \\ &\quad =\sum_{j=1}^K E_{\bar{S}_K,\bar{A}_K} \Biggl( \Biggl( \beta_0+ \sum_{k=1}^{K} \phi_k (S_k|\bar{S}_{k-1}, \bar{A}_{k-1}) - \tilde{\beta}_0 - \sum _{k=1}^{j} \tilde{\phi}_k (S_k|\bar{S}_{k-1}, \bar{A}_{k-1}) \Biggr) \\ & \qquad {}\times \bigl( g_j(A_{j}| \bar{S}_j,\bar {A}_{j-1}) - E_{A_{j}} \bigl(g_j(A_{j}|\bar{S}_j, \bar{A}_{j-1}) \bigr) \bigr) \Biggr), \end{aligned}$$

where we have again used the decomposition (5) to take the expectation over *Y*. The only terms involving *S*_*j*+1_,…,*S*_*K*_ are the $\phi_{k} (S_{k}|\bar{S}_{k-1}, \bar{A}_{k-1})$ which have expectation zero, so

This expression is equal to zero if the states are modelled correctly, i.e. if $\tilde{\phi}_{j}(S_{j}|\bar{S}_{j-1}, \bar{A}_{j-1})=\phi _{j}(S_{j}|\bar{S}_{j-1}, \bar{A}_{j-1})$ and $\tilde{\beta}_{0}=\beta_{0}$. Otherwise, if the treatment probabilities are modelled correctly then the expectation with respect to *A*_*j*_ gives zero because the first bracket does not depend on *A*_*j*_, and the expectation of the second bracket is zero.

### A.2 Consistency of $\hat{\psi}^{\mathit{RR}}$

We will show that *E*(*EE* ^*RR*^(*ψ*))=0 when the regret functions and the states have been modelled correctly, where

$$EE^{\mathit{RR}}(\psi) = \bigl(Y - E(Y|\bar{S}_{K}, \bar{A}_{K})\bigr) \sum_j \frac{\partial \mu_{j}}{\partial\psi}. $$

Let $\tilde{\phi}_{j}(S_{j}|\bar{S}_{j-1}, \bar{A}_{j-1})$ and $\tilde{\mu }_{j}(a_{j} |\bar{S}_{j}, \bar{A}_{j-1}\psi)$ be the postulated models for $\phi_{j}(S_{j}|\bar{S}_{j-1}, \bar{A}_{j-1})$ and $\mu_{j}(a_{j} |\bar{S}_{j}, \bar{A}_{j-1}\psi)$, respectively. Then the model for $E(Y|\bar {S}_{K},\bar{A}_{K})$ is

$$E(Y|\bar{S}_K,\bar{A}_K) = \tilde{\beta}_0 + \sum_{k=1}^K \tilde{\phi }_k(S_k|\bar{S}_{k-1}, \bar{A}_{k-1}) - \sum_{k=1}^K \tilde{\mu}_k (A_k | \bar{S}_k,\bar{A}_{k-1};\psi). $$

The expectation of the estimating equations over all random variables is then

$$\begin{aligned} & E_{\bar{S}_K,\bar{A}_K,Y}\bigl(EE^{\mathit{RR}}(\psi)\bigr) \\ &\quad = E_{\bar{S}_K,\bar{A}_K,Y} \Biggl( \Biggl( Y - \tilde{\beta}_0 - \sum _{1=2}^{j} \tilde{\phi}_k (S_k|\bar{S}_{k-1}, \bar{A}_{k-1})\\ &\qquad {}+ \sum _{k=1}^K \tilde{\mu}_k (A_k | \bar{S}_k,\bar{A}_{k-1};\psi) \Biggr) \sum_j \frac{\partial\tilde{\mu}_{j}}{\partial\psi} \Biggr) \\ &\quad = E_{\bar{S}_K,\bar{A}_K} \Biggl( \Biggl( \beta_0+ \sum _{1=2}^{K} \phi _k (S_k| \bar{S}_{k-1}, \bar{A}_{k-1}) - \sum _{k=1}^K \mu_k (A_k | \bar{S}_k,\bar{A}_{k-1};\psi) \\ &\qquad{} - \tilde{\beta}_0 - \sum _{k=1}^{j} \tilde{\phi }_k (S_k|\bar{S}_{k-1}, \bar{A}_{k-1}) + \sum _{k=1}^K \tilde{\mu}_k (A_k | \bar{S}_k,\bar{A}_{k-1};\psi) \Biggr) \sum_j \frac{\partial\tilde {\mu}_{j}}{\partial\psi} \Biggr), \end{aligned}$$

where we have used the decomposition (5) to take the expectation over *Y*. This expression is equal to zero if the regret functions and the states are modelled correctly, i.e. if $\tilde{\beta }_{0}=\beta_{0}$, $\tilde{\phi}_{j}(S_{j}|\bar{S}_{j-1}, \bar{A}_{j-1})=\phi _{j}(S_{j}|\bar{S}_{j-1}, \bar{A}_{j-1})$ and $\tilde{\mu}_{j}(a_{j} |\bar {S}_{j}, \bar{A}_{j-1}; \psi) = \mu_{j}(a_{j} |\bar{S}_{j}, \bar{A}_{j-1}; \psi)$.

## Appendix B: Uniqueness of the Regret-Regression Decomposition

The regret function is defined as

$$\begin{aligned} \mu_j(a_j | \bar{S}_j, \bar{A}_{j-1}) =& E\bigl(Y|\bar{S}_j, \bar {A}_{j-1}, \underline{d}_j^{\operatorname{opt}}\bigr)- E\bigl(Y| \bar{S}_j, \bar{A}_{j-1},a_j, \underline{d}_{j+1}^{\operatorname{opt}}\bigr) \\ = &E\bigl(Y|\bar{S}_j, \bar{A}_{j-1}, d_j^{\operatorname{opt}},\underline {d}_{j+1}^{\operatorname{opt}}\bigr)- E\bigl(Y|\bar{S}_j, \bar{A}_{j-1},a_j, \underline {d}_{j+1}^{\operatorname{opt}}\bigr). \end{aligned}$$

Suppose we wish to contrast the effect of action *A*_*j*_ with the best that can be achieved at time *j* on the assumption that rules *d* ^∗^ are followed in the future, not necessarily optimal. We might define

$$\mu_j^{\ast}(a_j | \bar{S}_j, \bar{A}_{j-1}) = E\bigl(Y|\bar{S}_j, \bar {A}_{j-1}, d_j^{\ast \operatorname{opt}},\underline{d}_{j+1}^{\ast} \bigr)- E\bigl(Y|\bar{S}_j, \bar{A}_{j-1},a_j, \underline{d}_{j+1}^{\ast}\bigr), $$

where $d_{j}^{\ast \operatorname{opt}}$ has the obvious interpretation. Suppose there is a corresponding nuisance function $\phi^{\ast}(S_{j} | \bar{S}_{j-1},\bar{A}_{j-1})$ and an equivalent decomposition to (5):

$$E(Y|\bar{S}_K,\bar{A}_{K}) = \beta_0^\ast+ \sum_{j=1}^K\phi_j^\ast(S_j | \bar{S}_{j-1},\bar{A}_{j-1}) -\sum _{j=1}^K\mu_j^{\ast}( A_j | \bar{S}_j,\bar{A}_{j-1}). $$

It must follow that

$$\begin{aligned} \begin{array}{c} \displaystyle\beta_0^\ast + \sum_{j=1}^K\phi_j^\ast(S_j | \bar{S}_{j-1},\bar{A}_{j-1}) \\ \\ \displaystyle\quad-\sum_{j=1}^K\mu_j^{\ast}( A_j | \bar{S}_j,\bar{A}_{j-1}) \end{array} \equiv \begin{array}{c} \displaystyle\beta_0 + \sum_{j=1}^K\phi_j(S_j | \bar{S}_{j-1},\bar{A}_{j-1})\\ \\ \displaystyle\quad-\sum_{j=1}^K\mu_j( A_j | \bar{S}_j,\bar{A}_{j-1}). \end{array} \end{aligned}$$

At time *K* there are no future actions. Hence $d_{K}^{\ast \operatorname{opt}}=d_{K}^{ \operatorname{opt}}$ and $\mu_{K}^{\ast}(A_{K} | \bar{S}_{K}, \bar{A}_{K-1})=\mu_{K}(A_{K} | \bar{S}_{K},\bar{A}_{K-1})$. Consequently

$$\begin{aligned} \begin{array}{c} \displaystyle\beta_0^\ast + \sum_{j=1}^K\phi_j^\ast(S_j | \bar{S}_{j-1},\bar{A}_{j-1}) \\ \\ \displaystyle\quad-\sum_{j=1}^{K-1}\mu_j^{\ast}( A_j | \bar{S}_j,\bar{A}_{j-1}) \end{array} \equiv \begin{array}{c} \displaystyle\beta_0 + \sum_{j=1}^K\phi_j(S_j | \bar{S}_{j-1},\bar{A}_{j-1})\\ \\ \displaystyle\quad-\sum_{j=1}^{K-1}\mu_j( A_j | \bar{S}_j,\bar{A}_{j-1}). \end{array} \end{aligned}$$

State *S*_*K*_ appears only in $\phi_{K}^{\ast}(S_{K} | \bar{S}_{K-1},\bar{A}_{K-1})$ on the left-hand side and only in $\phi_{j}(S_{K} | \bar{S}_{K-1},\bar{A}_{K-1})$ on the right-hand side. Since the equality holds for all *S*_*K*_, these terms must be identically equal. However, by definition $\phi_{K}^{\ast}(S_{K} | \bar{S}_{K-1},\bar{A}_{K-1})$ depends on following decision rule $d_{K}^{\ast}$ at time *K* whereas $\phi_{K}(S_{K} | \bar{S}_{K-1},\bar{A}_{K-1})$ assumes rule $d_{K}^{\operatorname{opt}}$ is followed. Thus, except in the special case of decisions having no effect, the decomposition can hold only if $d_{K}^{\ast}=d_{K}^{\operatorname{opt}}$.

We can continue in this way, successively cancelling terms, to show that a decomposition equivalent to (5) can hold only if $d_{k}^{\ast }=d_{j}^{\operatorname{opt}}$ for *j*=1,2,…,*K*.
